# Meeting Report on the Assisted Gene Flow and Climate Change Responses Workshop, Golden Gate National Recreation Area, CA, USA, 5–7 March 2025

**DOI:** 10.1111/eva.70290

**Published:** 2026-07-02

**Authors:** Lynn Breithaupt, Nicholas J. Kooyers, Jason P. Sexton, Benjamin K. Blackman

**Affiliations:** ^1^ Department of Life and Environmental Science University of California, Merced Merced California USA; ^2^ School of Biological Sciences University of Louisiana, Lafayette Lafayette Louisiana USA; ^3^ Department of Plant & Microbial Biology University of California, Berkeley Berkeley California USA; ^4^ Department of Integrative Biology University of California, Berkeley Berkeley California USA

**Keywords:** assisted gene flow, climate adaptation, conservation management, ecological restoration, evolutionary rescue, seed sourcing

## Abstract

Anthropogenic climate change is rapidly disrupting populations and ecosystems, challenging conservationists to identify effective interventions. Assisted gene flow (AGF), the human‐assisted movement of species within their historic range to increase the population health of climate‐threatened species, offers a promising but controversial strategy for enhancing resilience. To address the complexities of strategies for conserving species in anticipation of changing climates, we convened the Assisted Gene Flow and Climate Change Responses workshop from March 5–7, 2025, in Golden Gate National Recreation Area, California. The workshop was intended to be a forum for land managers, native seed producers, and academic scientists working in California and Oregon to discuss current and emerging knowledge of climate change responses, experiences, best practices, and policy associated with guiding ecologically informed applications of AGF in plants. The workshop brought together 28 participants, including land managers from five state or federal entities, native seed producers from two nurseries, and academic scientists from eight institutions. Through synthetic discussions, workshop participants identified major barriers to implementation, outlined critical outstanding questions, and proposed opportunities for coordination and joint action. The group reached an important consensus that while imperfect information remains, the risks of inaction are substantial, necessitating improved communication and immediate cross‐sector collaboration to support land and restoration management in effectively implementing these strategies. Moreover, the group highlighted how restoration projects are evolutionary experiments that can be leveraged to address outstanding fundamental and applied questions, and the critical need for the development of evidence‐based consensus guidelines to bridge the gap between research and application.

## 1 Introduction | Assisted Gene Flow: Promise and Debate in Climate Change Conservation

Climate change is disrupting populations, communities, and ecosystems (Calvin et al. 2023). A central challenge in conservation and restoration is determining how to allocate limited resources for maximum benefit in the face of these disruptions. Recognizing that effective solutions require cross‐sector collaboration, we organized the Assisted Gene Flow and Climate Change Responses Workshop at Golden Gate National Recreation Area in California in March 2025. The event engaged those working with natural plant populations in California and Oregon in an open dialogue about climate resilient conservation practices currently being implemented, considered, or constrained, including assisted gene flow. Discussions focused on current and emerging knowledge of climate change responses, experiences, best practices, and the policies associated with guiding assisted gene flow. By bringing together this diverse group, including environmental modelers, evolutionary ecologists, geneticists, seed producers, and land managers from state and federal agencies, we sought to share a broad exchange of perspectives on the risks and benefits of different interventions and to foster a greater understanding of each professional group's needs and incentives.

Foundational to these discussions was the recognition that describing climate change responses for a large variety of species is key for identifying and quantifying risks within communities and ecosystems (Iknayan and Beissinger 2018; Kooyers et al. 2025). A substantial literature suggests that many species are exhibiting declines within populations (Campbell 2019; Exposito‐Alonso et al. 2022; Inouye 2008; Krushelnycky et al. 2013; Reed et al. 2021; Sheth and Angert 2018), are becoming maladapted (Anderson and Wadgymar 2020; Browne et al. 2019; Kooyers et al. 2025; Wilczek et al. 2014; Wu et al. 2026), or have declining ranges or shifting phenology due to changing climates (Mills et al. 2013; Parmesan 2006; Parmesan and Yohe 2003). Although some species have been able to move to track their climatic niche, or have either acclimated or adapted to changing conditions (i.e., Tingley et al. 2009; CaraDonna et al. 2014), organismal responses are often challenging to determine as identifying potential responses involves substantial field work and logistically challenging experimental designs.

One conservation intervention for climate resilience considered highly promising by some but seen as concerning and in conflict with longstanding principles by others is assisted gene flow (AGF). We define AGF as the human‐assisted movement of species within their historic species range in order to increase the abundance or health of focal populations (Aitken and Whitlock 2013; see Section 2.3 for issues associated with defining AGF). AGF can increase genetic diversity in small populations to aid in the recovery from the deleterious effects of inbreeding and genetic drift, an impact known as genetic rescue (Beheregaray et al. 2026). Additionally, AGF can introduce alleles into focal populations that can help populations adapt to ecological stressors, an impact known as evolutionary rescue (Bell 2017; Carlson et al. 2014; Gomulkiewicz and Holt 1995; Orr and Unckless 2014). Both theoretical models and lab experiments suggest that AGF can rescue populations under certain circumstances (Browne et al. 2019; Encinas‐Viso et al. 2024; Grummer et al. 2022), and AGF has been employed in managing several threatened species with few other options for conservation (e.g., Fredrickson et al. 2007; Hagedorn et al. 2021; Hedrick 1995; Pregler et al. 2023). However, acceptance and adoption of AGF as a common practice has been uneven.

Many logistical questions about implementation and ethical concerns regarding potential consequences remain unresolved, creating the need for unified approaches and clearer guidelines, tools, and rubrics, although some efforts are emerging (see Carvalho et al. 2021; Silva et al. 2025; St. Clair et al. 2022). This lack of consensus creates a critical operational challenge: managers are often forced to operate in isolation. In the absence of prevailing evidence‐based agency or government guidelines, individual regions, forests, parks, and other units must make decisions at the local level with varying tolerances for risk. To bridge these gaps between theory and practical application, the workshop sought to synthesize fundamental knowledge about organismal responses to climate change with knowledge of logistical and technological hurdles facing native seed procurement, and in doing so, identify resources, tools, and venues that may facilitate greater collaboration and communication. Here, we report outcomes of these discussions, organized by three priorities identified as essential for unifying the field: (1) understanding the risks and barriers to interventions for climate resilience; (2) identifying the knowledge gaps that constrain action; and (3) forging pathways for meaningful collaboration between science and practice.

## 2 Threats, Barriers, and Risks to Climate Change Response Interventions

Despite the growing consensus that passive conservation strategies may be insufficient under rapid climate change, the implementation of active interventions such as AGF remains constrained by a complex array of impediments. Workshop discussions revealed that these obstacles span three distinct yet interconnected domains: scientific, practical, and sociological. First, scientific barriers involve the knowledge gaps associated with translocation uncertainties and risks, such as outbreeding depression and the unpredictability of ecosystem responses in novel environments. Second, practical barriers represent the operational constraints limiting preferred actions. Participants highlighted the impacts of diminished agency funding, staffing shortages, and significant logistical bottlenecks in the native seed supply chain, from procurement and propagation to storage. Finally, sociological barriers encompass the institutional and cultural challenges inherent in shifting conservation paradigms, including regulatory frameworks, communication deficits between researchers and land managers, and persistent ethical debates regarding the “local‐is‐best” sourcing standard (Jones 2013) versus the risks of inaction. Here, we lay out these multifaceted risks and the challenges they pose to developing unified, evidence‐based management strategies.

### 2.1 Scientific Barriers: The Uncertainty of Intervention

Among the barriers identified by workshop participants, scientific uncertainties were the most frequently discussed. These challenges span understanding genetic risks, ecological unpredictability, and the complexity of matching genotypes to future environments to create actionable management practices. While participants acknowledged concerns about potential risks from AGF, they emphasized that these theoretical predictions have rarely materialized at the scale or severity initially feared.

The actual magnitude of risk depends heavily on implementation details, particularly the proportion of introduced material relative to the recipient population size. One theoretical concern is genetic swamping: when climate‐adapted material is introduced in large quantities, abundant introduced genotypes could replace or dilute locally unique alleles rather than supplementing them (Laikre et al. 2010), potentially homogenizing genetic diversity and eroding cryptic local adaptations that have evolved over millennia to handle site‐specific stressors (Aitken and Whitlock 2013). However, participants noted that natural selection will likely favor genotypes with higher survival and fitness, potentially mitigating maladaptive outcomes. The key will be calibrating the introduction scale: low‐to‐moderate levels of gene flow may boost adaptive diversity without the risk of displacing locally adapted variants.

A related concern is outbreeding depression, which occurs when admixture occurs between distant populations or closely related species. Outbreeding depression can potentially break up co‐adapted gene complexes, groups of genes that have evolved to work well together. The resulting hybrid offspring may exhibit lower survival rates or impaired reproduction, essentially undermining the population the intervention was meant to bolster (Edmands 2007; Frankham 2015). Although the first generation after mating between different populations may show hybrid vigor as gene flow masks recessive deleterious alleles, these benefits can diminish over time as genomes recombine (i.e., hybrid breakdown). Conversely, transgressive segregation, where offspring exhibit extreme or novel phenotypes from gene flow may persist long‐term, creating new adaptive combinations. These genetic shifts may ripple through ecological networks, potentially altering competitive relationships, pollinator interactions, predator–prey dynamics, or plant–plant interactions in unpredictable ways, changes that could prove either beneficial or detrimental depending on the adaptive capacities of interacting taxa.

Despite these theoretical risks, evidence of negative effects from AGF is generally lacking; experimental studies suggest positive outcomes from gene flow outweigh negative ones (Kottler et al. 2021; Bontrager and Angert 2019). Meta‐analyses of genetic rescue interventions reveal that introducing genetic variations into small, inbred populations typically results in higher fitness outcomes (Bontrager and Angert 2019; Fitzpatrick et al. 2020; Frankham 2015; Pregler et al. 2023; Sexton et al. 2011). Nevertheless, the need for long‐term monitoring and additional studies on AGF's population and ecosystem effects remains clear (Bucharova 2017), including its integration into adaptive management frameworks for natural lands (Ashley et al. 2003; Williams et al. 2009).

Finally, barriers arise from the complexity of matching genotypes to future environments. Successful adaptation is not solely determined by tolerance to high temperature and aridity; it also relies on matching physiology to local edaphic conditions (Arenas et al. 2025) and on responding appropriately to myriad environmental cues for critical life history processes. Genetic mismatch of seed dormancy requirements, seasonal photoperiods, biotic interactions, or responses to local environmental cues may result in “climate‐matched” alleles failing (Lamont and Pausas 2023; Willis et al. 2014). For example, populations at the warm, trailing edge of a species' range can be valuable sources of heat‐adapted or drought‐adapted alleles and should be conserved as potential future sources of AGF with climate change (Hampe and Petit 2005; Sexton et al. 2011). Nevertheless, heat‐adapted seeds may not necessarily perform well in other parts of the range due to variation in local ecological factors mentioned above. However, seed mix strategies that combine local, regional, and distant genotypes are rarely tested and require further study (Bucharova et al. 2019). Yet even where scientific guidance exists, translating it into field practice presents its own distinct set of challenges.

### 2.2 Practical Barriers: Information, Resource, and Supply Chain Deficits

Beyond scientific uncertainties, workshop participants identified a set of practical barriers that constrain AGF implementation. Key operational questions such as how to select source populations, assess genetic risks, and evaluate success were acknowledged to lack widely accepted answers, leaving land managers without clear protocols (California Native Plant Society 2023; Flanagan et al. 2018; Ridley and Alexander 2016). Participants emphasized that these information deficits, compounded by resource scarcity and commercial supply chain realities, limit the on‐the‐ground capacity to execute AGF, even as predictions and demonstrations of its beneficial impacts continue building.

Chief among these information deficits is the scarcity of on‐the‐ground data from both source and recipient populations. Effective AGF benefits from knowledge of genotype‐by‐environment (G × E) interactions to predict future success, yet this information is rarely available or remains incomplete for many species. The “gold standard” for defining success, fitness‐related demographic increases demonstrated by multi‐year common garden experiments or provenance trials, is prohibitively costly and time‐consuming for the scale of restoration needs (Johnson et al. 2004; Walters et al. 2022). Genomic approaches offer a promising alternative for identifying seed source populations; however, their application within restoration remains largely academic and has yet to transition into standard operational practice (but see Buck et al. 2026; Fahey et al. 2025), even as agricultural sectors rapidly develop and integrate such tools into seed sourcing and adaptive breeding pipelines for crop improvement (Beck et al. 2025; Escamilla et al. 2025; Lezzi et al. 2026; Tyagi et al. 2024). Although a growing body of research employs common garden experiments across both perennial and annual plant systems (Kilkenny et al. 2026; Vicente and Benito Garzón 2024; Wu et al. 2026) and genomic diversity datasets are increasingly available (Olson et al. 2025; Schwacke et al. 2025; Shaffer et al. 2022), translating this knowledge into AGF practice remains limited, leaving land managers to make decisions under considerable uncertainty.

That uncertainty manifests differently across practitioners. The biological complexity of introducing novel genotypes raises unresolved questions about effectiveness and risk, leading some managers to default to local genotypes while others proceed with non‐local seed out of pragmatic necessity, satisfying budgets and deadlines without better information to inform their choices. For some, local fidelity is a matter of principle: Julia Michaels from Hedgerow Farms noted that most of her clients prefer local native seed sources, and some restrict collection to within a single zip code. Similarly, Eric Grijalva described how restoration at Golden Gate National Recreation Area relies primarily on seed collected within park boundaries, grown as nursery stock before replanting. Yet for others, pragmatic pressures override such preferences. Ed Kleiner from Comstock Seed Farm noted that seed demand for Bureau of Land Management restoration projects across California and Nevada is so high that managers sometimes request anything available, even if it did not originate from preferred seed transfer zones or climate‐matched areas. As a result, genotype mixing in restoration is already underway, often by necessity rather than design. Ironically, more may be known about the genetic outcomes of introductions from biological invasions (Dlugosch and Parker 2008) than from restoration reseeding, highlighting how the absence of systematic data collection leaves practitioners operating without the feedback loops needed to improve future decisions.

Workshop discussions highlighted that even where data exist, infrastructural barriers can silo these resources and prevent their use. Without centralized public repositories for genomic databases, published common garden research, and land manager field datasets, relevant data may remain fragmented and available only to specific agencies, academic institutions, non‐profits, or seed producers, preventing integration for cross‐sector use and perpetuating knowledge gaps that maintain the status quo. Thus, climate‐adapted restoration practices can remain undeveloped not because the necessary baseline data are absent, but because the infrastructure for all to connect and apply them does not exist.

Workshop participants also discussed how an acute erosion of organizational capacity also threatens AGF implementation. AGF demands considerable resources, including site preparation and ongoing post‐planting monitoring to assess evolutionary outcomes—challenges common to restoration projects broadly. However, the agencies tasked with this work are facing historic contractions in workforce and budget. Participants noted that recent fiscal constraints and personnel actions have devastated the continuity required for long‐term ecological projects and communication building. For example, recent reports indicate workforce declines of up to 39% in regions of the U.S. Forest Service (Mohr 2025), nearly 25% in the National Park Service (NPCA 2025), and a 23% reduction in EPA research staff (Daly 2025). Professional societies have warned that such attrition makes the sustained monitoring required for AGF effectively impossible (American Fisheries Society 2025). In this environment of scarcity, managers are often forced to prioritize immediate triage over complex planning for interventions like AGF. Even when AGF projects do proceed, the multi‐year monitoring to evaluate their success becomes less feasible, leaving critical questions about effectiveness unanswered.

Finally, the logistical realities of the commercial native seed sector present practical challenges. While prescriptive, genetically diverse mixes may be desired, the operational reality of native seed acquisition and amplification often necessitates compromise, depending on funding. For the forestry service, economic constraints typically favor procurement through seed‐transfer zones, a method that mixes seed from different parents of the same species within a zone defined by specific climate parameters (Erickson and Halford 2020) or by trait‐environment associations (Kilkenny et al. 2026). This approach, while cost‐effective, may not always achieve the level of genetic diversity across various climate zones that researchers envision (Clark et al. 2023; Walters et al. 2022).

The expense of maintaining genetic integrity in commercial settings creates further barriers. For example, Julia Michaels noted that Hedgerow Farms aims to maintain isolation distances between ecotypes of the same species during seed production. This practice requires careful spatial planning, long‐term crop placement strategies, specialized facilities, and dedicated labor, adding to the overall expense of the seed, and many operations cannot provide these services without guaranteed markets (Dadlani et al. 2023; Kumar et al. 2023; McCormick et al. 2021). Careful storage protocols add to these costs. Although mixing seed lots from different harvest years can reduce expenses and buffer supply, it risks introducing some maladaptive genes if the production years differ significantly in climatic selection pressures (Trusiak et al. 2023). Nevertheless, local natural selection can mitigate some degree of maladapted seeds, especially in more genetically diverse populations (Willi et al. 2022).

These challenges are compounded by inconsistent tracking and certification standards across the industry. Ed Kleiner noted that his work collecting seed for the Bureau of Land Management typically involves single‐source certification, origin validation, and purity and viability testing. However, certification requirements vary among federal and state agencies, impeding standardization of practices. Seed storage and mixing protocols for combining different years or genotypes lack industry‐wide standards entirely. Consequently, even if a land manager orders a climate‐resilient mix, one combining genotypes from multiple source populations selected for their predicted adaptive capacity under future conditions, the commercial pipeline may struggle to deliver it at the requested scale and genetic variety.

### 2.3 Sociological Barriers: Paradigms, Policy, and Communication

The practical barriers described above are in many ways tractable. Advances in genomic tools, improved data infrastructure, and restored institutional capacity could meaningfully address them over time. Yet workshop participants emphasized that even well‐resourced, technically equipped practitioners must navigate a distinct and often more intractable set of challenges rooted not in data or logistics, but in culture, communication, and governance. Land managers must contend with deeply held values about what constitutes “natural” restoration, bridge communication gaps between disciplines, operate within regulatory frameworks, and justify high‐stakes decisions with uncertain outcomes. These sociological and political barriers, rooted in philosophy, language, governance, and risk perception, shape whether, when, and how AGF is adopted, often exerting more influence over conservation outcomes than the biology itself.

Perhaps the most fundamental cultural tension centers on the question of “how local is local?” (McKay et al. 2005). Highly local philosophies prioritize the use of locally sourced genetic material in conservation, viewing geographic provenance as essential to ecological authenticity (Breed et al. 2018). This perspective stands in direct tension with AGF's core premise: that introducing non‐local genotypes may be necessary to enhance adaptive potential under climate change. In contrast, recent perspectives counter that healthy populations regularly receive alleles from other populations by natural processes and restoration practices should emulate these natural migration patterns (Sexton et al. 2024; Sgrò et al. 2011). The clash between these philosophies has real consequences. For instance, Christy Brigham noted how in (Wilderness Watch, Sequoia ForestKeeper, Tule River Conservancy, and John Muir Project v. National Park Service 2023), environmental groups sued to halt planting of Sequoia seedlings from outside their immediate groves in fire‐damaged wilderness areas, arguing this violated the Wilderness Act's protection of “untrammeled” genetic integrity. The National Park Service countered that extensive fire mortality makes natural regeneration insufficient. As rapid climate change drives more severe weather events, such conflicts place conservation land managers in a dilemma: choosing between adhering to traditional locality principles or responding to climate‐driven threats. This resulting tension and uncertainty can stall or delay plans or actions. However, pragmatic compromises exist, such as sourcing climate‐resilient seed within a relatively short, local radius (Buck et al. 2026) and leveraging the wide adaptive variation that may be found within and among populations across small spatial scales (Jahnke et al. 2025).

Even when philosophical alignment exists, communication failures can derail AGF initiatives. Scientists, land stewards, and community members operate with distinct professional vocabularies, leading to fundamental misunderstandings about concepts like “adaptation” or “success” (Amano and Berdejo‐Espinola 2025; Hannah et al. 2024). Even well‐established terminology carries different meanings across disciplines, and these semantic gaps can delay critical decisions when scientists attempt to communicate with policy stakeholders (Hannah et al. 2025). Surveys indicate that over 60% of land managers experience confusion or delays due to inconsistent translation of scientific findings (Sabo et al. 2024), contributing to a persistent evidence‐to‐policy gap (Rose et al. 2018). Terminology mismatches among agencies were highlighted during the workshop. For instance, while the U.S. Department of Agriculture categorizes AGF as assisted population migration to frame it as an extension of natural movement (Williams and Dumroese 2013), the National Park Service often classifies such interventions under the broader, more cautious umbrella of managed relocation (Schwartz et al. 2012). However, the choice of “migration” is more than a technicality; the term itself can act as a barrier to buy‐in, as it carries negative associations with the forced relocation of Indigenous peoples and is currently entangled in politically charged rhetoric regarding immigration policy.

Compounding semantic challenges are coordination challenges, including the absence of boundary‐spanning roles connecting different sectors, and organizational silos that prevent effective collaboration and communication (Safford et al. 2017; Sanders et al. 2021). These communication barriers are embedded within systems that were not designed for climate‐adaptive conservation. Most governance frameworks favor preservationist models that emphasize maintaining historical conditions rather than facilitating adaptation to new ones. Management, research, and educational permitting processes designed for traditional restoration can be rigid or ambiguous when applied to AGF, creating bureaucratic obstacles. For example, Raffica LaRosa outlined the permitting process for work involving endangered, threatened, or candidate California plant species and habitat types, under California Endangered Species Act and Federal Endangered Species Act regulations, noting that AGF research proposals require multiple reviews and are often met with heightened scrutiny due to concerns about unintended gene flow, loss of genetic diversity, and habitat encroachment. Although such caution is understandable given legitimate ecological concerns, these structural constraints can result in lengthy delays that postpone action as climate threats accelerate. Formal guidelines are greatly needed.

Together, these sociological barriers reveal that AGF is as much a social and political challenge as it is a practical and scientific one. Addressing these barriers requires deliberate investment in cross‐sector dialogue, cultivation of shared values and language, and structural reform of institutions to support climate‐responsive conservation strategies, pathways we explore in Section 4.

## 3 Bridging The Knowledge Gap: Outstanding Questions and Information Needs

Despite the substantial barriers outlined above, workshop participants arrived at a decisive consensus: the threat of climate‐driven population declines and ecosystem degradation far outweighs the theoretical risks of assisted gene flow interventions. Throughout the meeting, participants emphasized that inaction itself carries profound consequences while species are already experiencing range shifts, local extirpations, and declining fitness under current climate conditions. Concerns about AGF are frequently raised in the literature and among conservation practitioners, but participants noted that empirical evidence documenting negative outcomes remains scarce. In contrast, the mounting evidence of climate impacts on native populations demands an immediate response.

This sense of urgency was reinforced by workshop participants who described already implementing and testing climate‐adaptive strategies in their restoration and conservation work. Jason Sexton framed these interventions not as radical departures from natural ecological processes, but as deliberate enhancements of natural gene flow processes that recent research shows occur far more commonly than previously recognized (Sexton et al. 2024). Practitioners emphasized their need for evidence‐based frameworks to guide decision‐making and improve outcomes.

The workshop therefore focused on how to act strategically despite incomplete knowledge. Participants organized technical discussions around four outstanding questions that define the current boundaries of actionable science and highlight where targeted research can most rapidly inform practice: (1) How generalizable are species' responses to climate change? (2) How do we best predict which populations to use for AGF? (3) How do we perform AGF to maximize success? and (4) How do we define and measure success? In the sections below, we synthesize insights from workshop presentations and discussions to address each question, distinguishing what is known from what remains uncertain, and identifying the critical information needed to build robust, practical frameworks for climate‐adaptive conservation.

### 3.1 How Generalizable Are Species' Response to Climate Change?

Species responses to changing climates include moving, acclimating, and adapting to avoid population declines and extinction. Although some responses, such as shifts in range size and location, can be approximated through occurrence data and modeled over time, identifying evolutionary and demographic responses requires longer‐term field studies and experiments. For instance, Jill Anderson, keynote speaker of the workshop, described a decade‐long dataset in Drummond's rockcress, *Boechera stricta*, showing declining population growth rates in three of five common gardens along an elevation gradient, pinpointing critical agents of selection (Anderson et al. 2025). This work highlighted how such studies can help determine which agents and targets of selection are most critical for driving fitness in current and projected climates.

Because climate change alters multiple abiotic variables simultaneously (e.g., temperature, precipitation, CO_2_) while others remain constant (e.g., photoperiod), researchers are focused on developing tools to predict the specific variables associated with plant function. Jim Thorne and Ryan Boynton's presentation discussed developing hydroclimatic predictor variables using the Basin Characterization Model (Flint et al. 2021, 2013) to better approximate current patterns and future changes in water availability. Of course, how well projecting individual abiotic pressures relates to actual fitness characteristics on the ground remains an important research area. Kyle Rosenblad described how interactions between different climatic factors, such as drought and freezing, can have both positive effects (synergistic) or negative effects (antagonistic) for populations. In Lemmon's willow, *Salix lemmonii* , alleles providing freeze tolerance also conferred drought resistance (Rosenblad and Ackerly 2024). However, other alleles may impact multiple traits in non‐optimal ways (Etterson and Shaw 2001). Biotic interactions are often important to incorporate in predictions, and eco‐evolutionary dynamics are likely to influence evolutionary rescue (Ge et al. 2024). For instance, Christy Brigham (NPS) noted during discussions that substantial bark beetle‐induced mortality during giant Sequoia restoration efforts was not well correlated with standard climatic envelope modeling.

The frequency and severity of extreme events are likely to play an outsized role in influencing population dynamics compared to gradually shifting means. Although studies capturing population‐level responses to extreme events remain rare (Grant et al. 2017), workshop participants discussed results from resurrection‐based studies on the biological impacts of the historic 2012–2015 California drought (Kooyers et al. 2025, 2021). Additionally, Elsa Cleland (addressing California poppy) and Blair McLaughlin (addressing blue oak) reported that southern populations were better adapted to experimentally imposed drought conditions, suggesting they could represent valuable source populations for AGF.

Finally, these talks ignited discussion on the generality of the *targets* (i.e., the genes or traits) of natural selection under global change. An important question is whether the traits underlying historical local adaptation are the same targets that are important for adaptation to changing climates. Both Ren Hamm and Daniel Runcie suggested that allelic variation associated with climate‐linked variables is critical for AGF, but consensus was lower regarding how to incorporate information about phenotypic trait variation. Life history strategies and seed dormancy emerged as critical phenotypes; for example, the existence of a seed bank can predict population survival during extreme events, yet basic knowledge about seed bank diversity and duration is often lacking. Another emerging theme from the discussions was the importance of trade‐offs between potential trait strategies: plants that are good “stress tolerators” may not be able to escape extreme events through phenological shifts, suggesting that evolution toward one strategy may not be adaptive in future climates.

### 3.2 How Do We Choose Source Populations?

The agents (e.g., drought stress) and targets of selection discussed above inform the choice of source populations for AGF interventions. Participants discussed varied approaches to such decisions. One strategy involves using historical climate data to find source populations that match the current or predicted future conditions of the threatened site. Seed or climate transfer zones (Havens et al. 2015) and tools such as the Seedlot Selection Tool (St. Clair et al. 2022) offer helpful methodologies. Matthew Kling presented a seed selection webapp, *Seeds of Change*, that adds soil layers to increase predictive power, and integrates species distribution maps for all California native species. Jim Thorne presented a tool for setting operational priorities for collecting conifer cones based on current supply, current demand and estimates of projected demand informed by local assessments of climate risk (Thorne et al. 2025). Joe Stewart (*Climate Adapted Seed Tool*) discussed how incorporation of provenance study data within selection tools may aid in incorporating unforeseen selection pressures and stressors for species. His methods utilize both historical and contemporary climate data in combination with common garden datasets to prioritize threatened and donor tree populations from the lens of carbon sequestration. Similar seed transfer zones based off common gardens have been developed for herbaceous species (Kilkenny et al. 2026). As helpful as these tools are likely to be, participants also noted that these tools would benefit from and potentially improve through validation of fitness variation and evolutionary rescue in natural settings.

Additional options for choosing source populations include incorporating phenotypic and genetic information into predictive models, although it remains an open question how much either factor improves prediction accuracy for future climates. Workshop participants discussed what metrics would be most meaningful for different climate scenarios or life history strategies. For instance, should scientists prioritize selection of accessions with phenotypic means more closely optimized for future climates or variance in key ecological traits? Lines with greater plasticity might be preferred over lines with fixed, albeit adaptive, trait values. Likewise, participants considered in what circumstances neutral genetic diversity may be sufficient to predict fitness and when including climate‐associated SNPs may substantially improve outcomes. Resolving these questions will require field experiments specifically designed to evaluate the relative success of different predictive frameworks.

### 3.3 How Do We Perform AGF to Maximize Success?

Workshop contributors also highlighted how, even when managers are ready to use AGF, there remain several logistical knowledge gaps about how to implement AGF as a climate‐adaptive conservation strategy. Land managers desire clear guidelines on *how* and *when* to introduce individuals. A key uncertainty is frequency (Halford et al. 2025): is a one‐time influx sufficient, or do multiple introductions provide a higher likelihood of success? Some published guidelines suggest introducing ~5%–20% of the population (e.g., Hedrick 1995; Grummer et al. 2022), but this number likely depends on the life stage introduced and many other life history factors.

A landscape‐scale AGF experiment presented by Nic Kooyers (UL‐Lafayette) suggested that introducing seeds may be more effective than seedlings, as variation in dormancy or germination among seeds likely provides a continued influx of source alleles into the recipient population over multiple generations (Hinrichs et al. 2025). Beyond numbers and life stages, the genetic composition of the introduction is an important decision. How many source populations should be included, and in what proportion to the local population? Some guidelines exist (e.g., see Sgrò et al. 2011) and new frameworks are being developed (Tengstedt et al. 2026), but some species may require different guidelines. For instance, deleterious alleles from AGF are likely to be removed by purifying selection quickly in annual species, but are likely to persist for much longer in long‐lived perennials. Thus, potential seed sources for perennial species may require greater scrutiny. Introducing hybrid individuals (crosses between donor and local populations) could promote more rapid incorporation of source alleles (Pregler et al. 2023; Thavornkanlapachai et al. 2025). Although empirical experiments comparing these strategies in nature are needed, simulation work can be undertaken rapidly to develop the most feasible restoration designs (e.g., Grummer et al. 2022), including in a species‐specific manner (Black et al. 2024; Mead et al. 2024).

### 3.4 How Do We Define and Measure Success?

A final, critical question arose regarding how stakeholders define “success” for AGF and climate‐adaptive restoration. Success definitions vary by biological scale and stakeholder interest. For individual species, success is often defined by offspring production; however, as was discussed by Jill Anderson, long‐term persistence requires population growth rates to rise above replacement over many generations.

Defining success as a single population's offspring growth rate may involve tradeoffs at higher levels of organization (i.e., communities or ecosystems). For instance, increasing the abundance of a focal species could inadvertently decrease other community members or alter pollinator networks. Conversely, defining success via community diversity metrics or ecosystem services might result in the loss of local, rare, or diminutive species, and/or lead to biotic homogenization (i.e., the same common generalist species occurring everywhere replacing rarer species with an overlapping niche). The information needed to resolve these definitions does not stem solely from biological experiments, but from active communication of values and needs across stakeholders and society.

## 4 Opportunities for Coordination and Joint Action

A key aim of the workshop was describing pathways for researchers and land managers to advance AGF or other conservation strategies to address the likely impacts of climate change on local populations, taking into consideration the risks, challenges, and open questions outlined above. Across several synthesis sessions, participants worked to identify opportunities for coordination or joint action by which researchers, native seed producers, and land managers could all aid each other to translate high‐quality data into best practices and evidence‐driven policy. Participants were also asked to consider and prioritize what information is most needed, useful, or practical to help support local implementation or general policy development for these strategies. These conversations highlighted a wide variety of opportunities to enhance collaboration across professions and provided several clear action items or best practices. Identified opportunities range from leveraging datasets generated by long‐term monitoring or native seed contracts as fruitful resources for evolutionary research, to expanded modeling and experimental work to inform seed source selection for assisted gene flow, to means of reducing communication barriers and information lags between academic scientists and land managers.

### 4.1 Fruitful Resources for Evolutionary and Applied Research

Multiple opportunities for synergies in data collection and analysis emerged through the group's discussions. For instance, land managers noted that there are often long‐term observational datasets or time series of seed or other collections that are unpublished but could be of great interest to researchers looking to illuminate how population parameters, phenotypes, or genomes have evolved and tracked environmental change. Similarly, it was recognized that every restoration project and native seed contract is essentially an experiment in action that can be studied to assess adaptive evolutionary change as well as the success of different intervention practices or native seed mixes. In practice, sustained monitoring and comparisons across projects are challenging given limitations on personnel time and lack of standardized metrics for evaluating intervention success through time. Further, pre‐intervention baseline data may not be systematically collected. The group concluded that reaching a consensus on what metrics of success may be most informative, while also imposing the least metadata or sample collection burdens, would be a potentially transformative short‐term goal to advance the field.

Finally, even as field experiments and other phenotypic surveys will continue to require high investments of effort, reference genomes and genotypic data are ever‐increasingly straightforward and affordable to obtain at scale, albeit requiring specific expertise to produce. For instance, the California Conservation Genomics Program supported resource generation and research advances by several of the workshop participants (Martínez‐Gómez et al. 2025; Nguyen et al. 2025; Shaffer et al. 2022; Toews et al. 2025). Thus, genomic datasets for examining demography, gene flow, and local adaptation that were once practically limited to a small number of model systems are now accessible for a greater range of taxa, including those that are more ecologically relevant or in demand as foundational or threatened species for particular ecosystems. The group saw high potential value in the identification of useful new regional study systems through communication between researchers, native seed producers, and land managers.

### 4.2 Recommended Practices

Another clear recommendation that emerged from the synthesis sessions was for the research community to develop and publish a strong set of evidence‐based guidelines for AGF and related conservation and restoration practices, and to update these guidelines regularly. Land managers emphasized that having published guidelines in the scientific literature would serve as an essential foundation that could then kickstart more effective policy development, including the establishment of predictive seed zones, best practices, and certification procedures. Native seed providers expressed that guidelines would also help inform the composition of native seed mixes, moving the industry toward stronger evidence‐based practices that can then effectively synergize with market needs and allow important seed pools to be prioritized for collection and production.

As discussed above, there are gaps between current knowledge and the body of evidence that would ideally be in hand to inform a document of consensus guidelines. Additional modeling and experimental work will be helpful in filling in these gaps. For instance, workshop participants highlighted how simulation studies and field experiments can complement each other to set practical decision parameters like how frequently and extensively to sample native seed from source populations, how much seed to introduce relative to the recipient population's size, or how to balance those introduced seed mixes with seed from multiple source populations. As the literature on this topic continues to grow rapidly, we expect that the time when an initial consensus around AGF guidelines could be achieved will nevertheless soon be here for at least some groups of species.

### 4.3 Improving Communication by Reducing Barriers and Information Lags

Ultimately, improving the frequency and ease of communication between professional groups is essential, and the workshop reinforced how important and beneficial it is when researchers and land managers are communicating. Each group—researchers, native seed producers, and land managers—has information needed by each other group, but barriers or information lags caused by terminology, resource constraints, or gaps in professional networks can impede trust, progress, and innovation. Likewise, within professional groups, communication can be siloed within institutions, divisions, agencies, or states, potentially limiting what intervention strategies are adopted. Ezra Kottler (University of the Pacific) presented results from a recent survey of restoration land managers working in California showing that meeting deliverables within the timelines and budgets established by grant support often necessitated reliance on commercially available native seedstocks. Insufficient time and resources often made new, project‐specific native seed collection and amplification efforts infeasible. Thus, building strong regional networks of native seed users and stronger pipelines of sources, whether production nurseries or managers of wild populations, would allow for the improved enhancement of species and genetic diversity in restoration projects as well as help advance strategies for climate resilience. Continued support for programs such as the U.S. Geological Survey Climate Adaptation Science Centers and U.S. Department of Agriculture Climate Hubs is essential to overcoming communication barriers and facilitating connections among stakeholders, as well as hosting data and resource clearinghouses. Such regional networks would also provide stability and resilience in the face of an increasingly turbulent political landscape.

Land managers emphasized that government subscriptions to scientific journals are limited, and that this constraint significantly limits or delays what enters their sphere of awareness. Therefore, uploading manuscripts to preprint servers and open access publication are important and easily obtainable steps that academic researchers can take to ensure their work is in an effective venue to reach this audience. Digesting findings from one's work and related papers into a whitepaper with accessible language or going straight to land managers, for instance by offering to present at division meetings, was also strongly encouraged. As native seed production by commercial nurseries is an expanding agricultural enterprise serving our nation's public and native lands, communication between researchers and land managers could be greatly facilitated by land‐grant institutions hiring new Cooperative Extension faculty members focused on this boundary‐spanning area. As a core part of their work, these faculty would seek to translate university research on AGF and other climate change adaptation strategies into practical, science‐based information for native seed nurseries and the clients that contract with them. Finally, cross‐cutting workshops like the one we reviewed here can be excellent means for network building and open, productive, and synthetic discussions.

## 5 Conclusion | Aligning Science With Reality

The discussions at this workshop illuminated a practical truth: AGF is not merely a future consideration but an active, ongoing component of land management. Important goals are to improve, inform, and increase its use. As participants noted, restoration land managers are frequently compelled to make immediate decisions based on commercially available seed stocks, meaning that genetic movement is often occurring by default rather than by design. This “de facto” AGF underscores the urgent need to align operational necessity with scientific evidence. Without guidance, genetic mixing will continue to be dictated by supply chain availability rather than climatic or biological information that maximizes adaptive and conservation potential. The consensus among participants is that the “do no harm” paralysis driven by scientific uncertainty is increasingly untenable, as it risks becoming a decision to allow extirpation by default.

To bridge this gap, workshop participants suggested we should transition from conservation practices primarily guided by preservation‐minded, risk avoidance philosophies to adaptive stewardship. The consensus at the end of the workshop was that we cannot wait for perfect data before acting. Instead, we must approach restoration projects as evolutionary experiments. By embedding monitoring and adaptive management into ongoing restoration projects led by workshop participants including the National Park Service, the National Forest Service, and California Department of Natural Resources, land managers can generate the missing knowledge on implementation success that the field is needing. We found that regional workshops can serve as critical infrastructure for this transition, fostering a level of coordination between native seed producers, academics, and agency managers that is rarely achievable through publications or disciplinary conferences alone.

Ultimately, successful implementation depends on establishing traditions and institutionalizing these connections. We conclude with a call to action: we must move beyond debating the theoretical risks of AGF and focus on guiding implementations that are deliberate, evidence‐based, and communicated effectively. This requires a unified voice to advocate for the sustained funding and workforce stability needed to monitor these interventions. By embracing a framework of “learning by doing” and strengthening cross‐sector collaboration, we can equip our ecosystems with the resilience needed to survive in a rapidly changing world.

## Funding

This work was supported by the National Science Foundation (Grants IOS‐2222464, IOS‐2222466, IOS‐2222467, and DEB‐2045643).

## Conflicts of Interest

The authors declare no conflicts of interest.

## Data Availability

Data sharing not applicable to this article as no datasets were generated or analyzed during the current study.
